# Stigmatization and Social Support of Pregnant Women With HIV or Syphilis in Eastern China: A Mixed-Method Study

**DOI:** 10.3389/fpubh.2022.764203

**Published:** 2022-03-11

**Authors:** Xiaohui Zhang, Xiaoyan Wang, Hong Wang, Xinmei He, Xinyu Wang

**Affiliations:** ^1^Department of Women's Health, School of Medicine, Women's Hospital, Zhejiang University, Hangzhou, China; ^2^Department of Nutrition and Food Hygiene, School of Public Health, School of Medicine, Zhejiang University, Hangzhou, China; ^3^Department of Obstetrics, School of Medicine, Women's Hospital, Zhejiang University, Hangzhou, China; ^4^Department of Women and Children's Health, Women and Children Health Care Hospital of Quzhou, Quzhou, China; ^5^Women's Reproductive Health Key Laboratory of Zhejiang Province, Department of Gynecology and Obstetrics, School of Medicine, Women's Hospital, Zhejiang University, Hangzhou, China

**Keywords:** HIV, syphilis, pregnancy, stigmatization, social support, eastern China

## Abstract

**Background:**

Stigmatization and poor social support are challenges faced by individuals living with HIV or sexually transmitted disease, which can have a profound negative impact on their healthcare. Mother-to-child transmission of either HIV or syphilis can lead to adverse maternal and fetal outcomes. The aim of this study was to investigate stigmatization and social support of pregnant women with HIV or syphilis in eastern China.

**Methods:**

This was an explanatory sequential mixed-method study conducted in Zhejiang province, China in 2019. Stigmatization, social support, and the associated factors toward HIV or syphilis were evaluated using questionnaires. The social support rating scale was used to evaluate social support, where a score <25% was defined as poor social support. A logistic regression model was used to explore the association between stigmatization and poor social support.

**Results:**

A total of 448 women (HIV positive, *N* = 93; syphilis, *N* = 355) were recruited in this study. Higher stigmatization was observed in pregnant women with HIV compared to those with syphilis (53.76% vs. 24.36%, *p* < 0.001), and poorer social support was observed in women with HIV compared with those with syphilis (40.86% vs. 19.86%, *p* < 0.001), with significant distributions of the total social support scores (Z = −1.976, *p* = 0.048) and scores on objectivity (Z = −2.036, *p* = 0.042) and subjectivity (Z = −2.500, *p* = 0.012). Similar social support among HIV or syphilis pregnant women was observed in medical healthcare facilities. In multivariable logistic model analysis, stigmatization (OR_*adj*_ = 2.927; 95%CI, 1.714–4.996; *p* < 0.001) and ethnic minority (OR_*adj*_ = 2.373; 95%CI, 1.113–5.056; *p* = 0.025) were negatively associated with social support. Interestingly, employment status was associated with improved social support (OR_*adj*_ = 0.345; 95%CI, 0.180–0.662; *p* = 0.001).

**Conclusion:**

Stigmatization among pregnant women with HIV or syphilis remains high. We demonstrated that stigmatization was a significant predictor of low social support in pregnant women with HIV or syphilis. The support shown in medical facilities was similar toward pregnant women with HIV or syphilis. Implementation of stigmatization eradication and social support strategies targeting pregnant women with HIV or syphilis may therefore improve the dual elimination of mother-to-child transmission service.

## Introduction

Mother-to-child transmission (MTCT) of either HIV or syphilis is strongly associated with stillbirth, preterm birth, low birth weight, neonatal death, and congenital infection in infants (1, 2). The dual elimination of MTCT (EMTCT) of HIV and syphilis has been given a public health priority worldwide and is advocated by the World Health Organization (WHO), requiring over 95% of pregnant women to receive antenatal health care (ANC), HIV and syphilis screening, and treatment for positive women (3). Ensuring equal human rights and EMTCT services for women with HIV or syphilis are highlighted through awareness of stigmatization against HIV or syphilis (4).

HIV-related stigmatization is common, and it has been reported that 60–80% of HIV-infected adults have made at least one statement regarding HIV-related stigmatization (5, 6). People living with HIV (PLWH) face internalized or overt stigmatization, such as being ashamed of HIV infection, being the subject of malignant gossip, and being discriminated against in their living settings (5–8). Previous studies have shown that women have higher odds of experiencing both higher enacted stigmatization and internalized stigmatization than men at home and abroad (8–10). HIV-related stigmatization might discourage pregnant women from seeking and adhering to ANC (11–13). Women with syphilis experience stigmatization and discrimination in a manner similar to or even worse than those with other STDs (14, 15). Studies on HIV-related stigmatization were primarily conducted in a high HIV prevalence or poor resource setting; however, few studies have been conducted to explore syphilis-related stigmatization in pregnant women (16). Social support, especially perceived social support, is influential in promoting the maintenance of positive health behaviors among people living with HIV (17–19). For example, a study in the mid-west United States showed that PLWH who lack social support experience challenges in adhering to their treatments (17). Stigmatization associated with poor social support can lead to psychological distress, which may constitute a barrier to the implementation of EMTCT services (16, 19, 20). In addition, social support was revealed to be insufficient to improve health in PLWH who experienced HIV-related stigmatization (6). Therefore, it is important to explore the association between stigmatization and social support.

China is a country with an immense population and contains tremendous ethnic diversity, and the association between stigmatization and social support among pregnant women with HIV or syphilis remains poorly understood. Previous studies showed that HIV-positive pregnant women experienced more psychological distress and stigmatization than men, and HIV-related stigmatization was negatively associated with social support (9, 20–22). A majority of these studies in China focused on women living in HIV epidemic areas. Zhejiang province is located in eastern China, and it is economically well-developed, thereby attracting a large number of people from other provinces. Although, the prevalence of HIV in pregnant women is low in Zhejiang Province, Zhejiang faces a huge burden of maternal syphilis. In 2016–2019, Zhejiang ranked in the top-three areas of China for endemic syphilis, with a large number of pregnant women with syphilis (23). In 2017, Zhejiang was selected as a pilot province for the EMTCT program in China. The aim of the study was to draw a comprehensive understanding of HIV- and syphilis-related stigmatization and social support during pregnancy, and to explore the differences between HIV and syphilis-related stigmatization. Such information would assist in implementing targeted policies and strategies for EMTCT.

## Materials and Methods

### EMTCT Program

The EMTCT program in Zhejiang province offers HIV- and syphilis-screenings free-of-charge at the first ANC visit, and repeated testing in the third trimester or before child delivery. HIV diagnosis is determined at the local Center for Disease Control and Prevention. Maternal syphilis is confirmed by obstetricians at the local hospital when a woman has positive results for both non-treponemal and treponemal tests. The HIV- or syphilis-positive woman is then offered combined ANC and EMTCT services to ensure safe delivery at qualified health facilities. All infected women and their children were followed-up by the local Women and Children's Hospital. Data were collected using a national web-based reporting system.

### Study Design and Data Collection

An explanatory sequential mixed-method research study was conducted. Women were recruited at a local Women and Children's Hospital in Zhejiang province from January to December 2019. We included women with HIV or syphilis in the study. Women with HIV, syphilis, HBV, or other sexually transmitted diseases as co-infection during pregnancy were excluded. We initially collected quantitative data using a structured questionnaire at the first ANC to learn HIV- or syphilis-related stigmatization, social support, and associated factors.

We performed a convenience sampling. The sample size for the quantitative study was estimated by a simple random sampling method. According to the formula, the total numbers of pregnant women with HIV and syphilis (*study population* = *N*) were estimated as 100 and 3,000, respectively by annual epidemic estimation in Zhejiang. We estimated stigmatization incidence (*p*) in infected women to be 50%, with an allowable error (δ) and a significance level (α) of 0.05. Thus, the minimal sample for women with HIV was 82 and for women with syphilis was 340. Considering potential data missing, we enlarged the sample size by 10%.

Formula:


$$\begin{array}{l} n=\frac{Z_{\alpha /2}^{2}p\left(1-p\right)N}{\delta^{2}\left(N-1\right)+Z_{\alpha /2}^{2}p\left(1-p\right)N} \end{array}$$

In this study, 457 women were interviewed. Three women with HIV and syphilis coinfection along with six women who did not report their infection status as HIV or syphilis were excluded. Finally, 448 women participated in the study. Of them, 399 women met in face-to-face investigations with medical staff. A total of 49 women completed questionnaires online by themselves, and these were then evaluated by medical staff. Then, the medical staff checked the data for completeness and coherence.

We conducted a qualitative study to understand profound stigmatization and social support in one of the regions in Zhejiang province. Face-to-face interviews were conducted by trained local maternal and child healthcare providers for three women using an in-depth, semi-structured questionnaire. The participants were interviewed in a private room after completing their routine ANC at a local hospital, with the interview lasting 20 min on average per person. We used a convenience sampling method, and the total number of responds were decided through discussion with local maternal and child-healthcare providers. We provided a 15 USD gift for each participant as compensation for transportation at the end of the interview. Verbal data were audio-recorded and transcribed into written form simultaneously by study investigators. The themes were manually selected, which were internally coherent, highlighted, and coded. The contents were relevant to the study, including their socio-demographic information, first ANC visit experiences, barriers to EMTCT services, and the first time the individuals were informed of their HIV or syphilis infection status. The analysis team members reviewed the code, and the themes were given equal attention when being interpreted. There were three themes: theme 1, disclosure of infection status; theme 2, experience of stigma; and theme 3, support and barriers to EMTCT. Ultimately, convergence/triangulation of qualitative and quantitative was executed by two investigators.

With regard to the study, informed consent was provided by each participant, and the ethical clearance was obtained from the Ethics Committee of the Women's Hospital School of Medicine, Zhejiang University (No. 20180180).

### Measurements and Data Analysis

For this study, we estimated HIV- or syphilis- related stigmatization in women with HIV or syphilis for at least one of the following three items; living setting, workplace, and healthcare facility, in the past year. The social-support condition was assessed by the 10-item social support rating scale (SSRS) that contained 3 questions on objective support, 4 on subjective support, and 3 on the utilization of social support. We counted the total item scores of the SSRS and scores by subgroups. Objective support focused on the support resources and living area; while subjective support reflected close relationships with friends, family members, colleagues, and neighbors. The manner in which women asked for assistance and involvement in social activity revealed the utilization of social support. Higher scores indicated better social support.

The SSRS is widely used to investigate the social support in people living with HIV/AIDS, and in people with sexually-transmitted diseases in China (20). The study described the validity and reliability of using SSRS questionnaire in the Chinese population (24). It showed the Cronbach's α total scale to be 0.821 and the factors were 0.631–0.685; the split-half correlation coefficient for the total scale was 0.875 and the factors were 0.547–0.709; and the test–retest correlation coefficient was 0.829 and the factors were 0.645–0.756. With respect to the findings in this study, we used qualitative analysis to better understand and describe participants' opinions on their experiences of stigmatization and social support.

We recorded data using Excel software (Microsoft Excel; Microsoft Corp, Redmond, WA) and performed statistical analysis with SPSS (version 24.0, IBM Corp, 2016). Floating population in the study indicated that women came from other provinces to Zhejiang. With regard to quantitative data, descriptive analyses were conducted by mean and standard deviation (SD), median and interquartile range (IQR), frequency, and percentage. Poor social support was regarded as the lowest 25% of the total social support scores according to IQR. *T*-tests or Chi-Square tests were used to compare socio-demographic characteristics, distributions of support scores, and resources between pregnant women with HIV or syphilis. Non-parametric tests as Mann–Whitney U-tests were used for non-normal distribution variables. Logistic regression model was used to determine associations between significant factors with poor support. Main variables were defined as follows: poor social support (yes = 1, no = 0), maternal age group (aged ≤ 20 years as a reference grou*p* = 1, aged 21–39 years = 2, aged 30–34 year = 3, aged ≥ 35 years = 4), education (primary education as a reference grou*p* = 1, middle level education = 2, at or beyond college level education = 3), parity (nulliparous = 0, multipara = 1), maternal infection status (HIV infection = 1, syphilis infection = 0), employment (fixed employed = 1, unemployed = 0), floating population (yes = 1, no = 0), minority (ethnic minority = 1, Han nationality = 0), negative influence due to transportation (yes = 1, no = 0), negative influence due to economy (yes = 1, no = 0). A two-tailed *p*-value below 0.05 was considered statistically significant, unless otherwise indicated.

## Results

### Basic Characteristics of Study Participants

The participants comprised 93 pregnant women with HIV and 355 with syphilis. The mean maternal age was 29.23 ± 6.51 in pregnant women with HIV, which was similar to that for women with syphilis 29.87 ± 6.01 (*t* = 0.001, *p* = 0.372). There were no significant differences in the distribution of maternal age groups, educational level, employment, gravidity, parity, number of children, or partner's infected status between women with either HIV or syphilis (Table 1). HIV-positive women were more likely to be from a floating population, a minority, and were more likely to be diagnosed after the first trimester relative to women with syphilis infection (all *p* < 0.05). For HIV-positive pregnant women, 62.37% came from outside Zhejiang Province, 19.35% were from a minority group, and 55.43% knew their infection status before pregnancy. Local residents accounted for 52.39% of women with syphilis, the minority proportion was only 9.30%, and the majority was identified in the first trimester. The mean gestational age at the first ANC visit was comparable in HIV- (11.96 ± 7.07 weeks) and in syphilis-positive (12.42 ± 5.93 weeks) pregnant women (*t* = 1.902, *p* = 0.549).

**Table 1:** Comparison of basic characteristics between women with HIV and syphilis.

**Variable**	**HIV (*N* = 93)**		**Syphilis (*N* = 355)**		**χ^2^**	***p***
	***N***	**%**	***N***	**%**		
**Maternal age (years)**							
≤ 20	8	8.60	16	4.51	3.353	0.340
21–29	40	43.01	160	45.07		
30–34	27	29.03	91	25.63		
≥35	18	19.35	85	23.94		
**Education**						
Primary school	12	13.04	49	13.80	4.649	0.199
Junior middle school	36	39.13	177	49.86		
High school	28	30.43	88	24.79		
College	16	17.39	41	11.55		
Missing data	1	/	/	/		
**Living area**							
Local	35	37.63	186	52.39	7.224	0.027
Floating population	58	62.37	169	47.61		
**Minority**							
Han	75	80.65	322	90.70	6.021	0.013
Others	18	19.35	33	9.30		
**Employment**							
Employed	65	69.89	253	71.27	0.068	0.795
Unemployed	28	30.11	102	28.73		
**Gravidity**							
1	20	21.97	70	20.00	1.347	0.510
2	25	27.47	80	22.86		
≥3	46	50.55	200	57.14		
Missing data	2	–	5	–		
**Parity**							
0	27	30.34	108	30.59	0.002	0.962
≥1	62	69.66	245	69.41		
Missing	4	–	–			
**Number of Children**							
0	33	36.26	118	33.33	0.277	0.598
≥1	58	63.74	236	66.67		
Missing data	2		1			
**Partner's infection**							
Yes	65	69.89	220	61.97	1.997	0.158
No	28	30.11	135	38.03		
**Timing of diagnosis**							
Before pregnancy	51	55.43	100	28.33	29.640	p <0.001
First trimester	26	28.26	158	44.76		
Second trimester	7	7.61	72	20.40		
Third trimester	4	4.35	17	4.82		
After delivery	4	4.35	6	1.70		
Missing data	1		2			

*Missing data were excluded*.

### Stigma and Social Support

Overall, 53.76% (50/93) of women with HIV and 24.36% (86/353) of women with syphilis experienced stigmatization (χ^2^ = 30.413, *p* < 0.001) in the past year. Around 40.86% (38/93) of HIV women admitted to low social support, which was also markedly higher than women with syphilis (19.83%, 70/353; χ^2^ = 17.739; *p* < 0.001). The distribution of the social support total scores (Z = −1.976, *p* = 0.048), objectivity scores (Z = −2.036, *p* = 0.042), and subjectivity scores (Z = −2.500, *p* = 0.012) significantly differed between HIV-infected pregnant women and those with syphilis infection (all *p* < 0.05) (Table 2).

**Table 2:** Scores of social support between women with HIV and syphilis.

**Valuable**	**HIV**		**Syphilis**		**Z**	***p***
	**Median**	**IQR**	**Median**	**IQR**		
Scores	31.00	23.00–41.5	34.00	28.00–41.00	−1.976	0.048
Objective	4.00	4.00–13.00	8.00	4.00–12.00	−2.036	0.042
Subjective	19.00	15.00–22.25	20.00	16.00–24.00	−2.500	0.012
Utilization	6.00	5.00–7.00	6.00	5.00–8.00	−0.609	0.542

The distributions of support by economy and transportation differed significantly between women with HIV and syphilis (all *p* < 0.05). The social impacts from friends and families were almost similar; however, we noticed no significant impact from medical staff (*p* = 0.932) (Table 3). In multivariable logistic analysis, poor social support was frequently observed in women from minority ethnic groups compared to women of the Han ethnicity (OR_*adj*_ = 2.373; 95%CI, 1.113–5.056; *p* = 0.025), in women who experienced stigmatization compared to those who did not (OR_*adj*_ = 2.927; 95%CI, 1.714–4.996; *p* < 0.001), and in women with HIV compared to women with syphilis (OR_*adj*_ = 2.132; 95%CI, 1.213–3.747; *p* < 0.001). Employment was a favorable factor for social support (OR_*adj*_ = 0.345; 95%CI, 0.180–0.662; *p* = 0.001) (Table 4).

**Table 3:** Distribution of social-support scores.

**Value**	**HIV**		**Syphilis**		**χ^2^**	***p***
	***N***	**%**	***N***	**%**		
Economy	Strong	28	30.11	187	52.97	15.16	0.001
	Medium	52	55.91	139	39.38		
	Weak	11	11.83	24	6.80		
	Unknown	2	2.15	3	0.85		
Transportation	Strong	44	47.31	234	66.29	9.74^*^	0.008
	Medium	38	40.86	102	28.90		
	Weak	7	7.53	13	3.68		
	Unknown	4	4.30	6	1.70		
Friends and family	Strong	37	39.78	171	48.44	6.22	0.045
	Medium	29	31.18	132	37.39		
	Weak	14	15.05	27	7.65		
	Unknown	13	13.98	22	6.23		
Medical staff	Strong	44	47.31	157	44.48	0.142	0.932
	Medium	24	25.81	86	24.36		
	Weak	23	24.73	91	25.78		
	Unknown	2	2.15	18	5.10		

*Women no response were excluded*.

* *Chi-square correction*.

**Table 4:** Associated factors with poor social support for infected women.

**Variable**	**OR**	**Wald**	**df**	***p***	**OR 95% CI**	**OR**	**Wald**	**df**	***p***	**OR _(adj)_ 95% CI**
**Age**											
≤ 20 (ref)		8.706	3	0.033			3.774	3	0.287	
21–29	0.932	3.960	1	0.407	2.538 (1.014–6.354)	0.560	0.885	1	0.347	1.751 (0.545–5.630)
30–34	0.095	0.119	1	0.730	1.100 (0.641–1.889)	−0.068	0.041	1	0.839	0.934 (0.484–1.803)
≥35	−0.432	1.705	1	0.192	0.649 (0.340–1.241)	−0.476	1.660	1	0.198	0.622 (0.302–1.281)
**Education**											
Primary (ref)	–	3.537	2	0.171	–	–	5.034	2	0.081	
Middle	−0.508	3.036	1	0.081	0.602 (0.340–1.066)	−0.802	2.246	1	0.134	0.449 (0.157–1.280)
High	−0.187	0.232	1	0.630	0.829 (0.387–1.778)	−0.899	5.016	1	0.025	0.411 (0.189–0.895)
Floating	0.460	4.458	1	0.035	1.584 (1.034–2.429)	0.203	0.530	1	0.467	1.225 (0.710–2.114)
Minority	0.923	9.093	1	0.003	2.518 (1.382–4.589)	0.892	5.008	1	0.025	2.373 (1.113–5.056)
Employed	−0.993	12.473	1	<0.001	0.371 (0.214–0.643)	−1.065	10.232	1	0.001	0.345 (0.180–0.662)
Parity	−0.428	3.479	1	0.062	0.652 (0.415–1.022)	−0.494	3.239	1	0.072	0.610 (0.357–1.045)
Stigma	1.065	22.488	1	<0.001	2.901 (1.868–4.506)	1.074	15.485	1	<0.001	2.927 (1.714–4.996)
Infection	0.990	16.173	1	<0.001	2.692 (1.661–4.362)	0.757	6.918	1	0.009	2.132 (1.213–3.747)
Transportation	0.407	3.449	1	0.063	1.502 (0.978–2.308)	−0.075	0.060	1	0.807	0.928 (0.509–1.692)
Economy	0.342	2.481	1	0.115	1.408 (0.920–2.157)	−0.139	0.204	1	0.651	0.871 (0.477–1.589)
Constant	–	–		–	–	0.132	0.021	1	0.885	–

### Qualitative Analysis

Two pregnant women with HIV and one with syphilis underwent a detailed interview. All of them possessed a middle-school education and were 23–31 years of age. One woman with HIV infection was a local resident from Zhejiang Province, while the other two were not. Two women were interviewed during their ANC, while one was interviewed during her postpartum healthcare visit. The participants described their first-time experience and perspectives when they learnt about their HIV or syphilis status, and when they attended their first ANC visit.

“*When my doctor told me I had HIV infection, my mother and **I felt terrible** because the doctor said HIV increased the risk of death. Later, I met my husband, who is also HIV positive. We supported each other and adhered to treatment. I came to this city with my husband. I trust my doctor at the Women and Children's Hospital. We were scared that the baby would be infected as well. A doctor in the ANC department told me that if I received EMTCT service, the risk of our baby getting infected would be reduced. **The doctor was kind to us. I felt warm, because she gave me her mobile phone number**. ” (Woman, with HIV, 29 years old, middle-school education, first pregnancy, at 24 gestational weeks.)*

“*I was diagnosed with HIV during my first ANC visit. **I noticed some doctors to be unfriendly** for they whispered. I told my husband about my disease immediately. He also received HIV screening, which came back negative. However, my husband did not blame at me and helped me a lot. I live in a rural area. I don't go out much because I'm afraid people will know about my HIV status. **I have no friends**. I hope to have a platform for communication.” (Woman, with HIV, 31 years old, middle-school education, postpartum, with a baby)*

“*Two years ago, I was informed that I was infected with syphilis when I went to a dermatologist. My husband was also syphilis-positive. **I was ashamed of it**. I learned online that syphilis infection is associated with sexually transmission, but both of us do not have unsafe sexual behaviors. I could not understand why I was infected. My friends do not know about this and I won't tell my friends about it. This might be due to underwear being contaminated. **Most of the medical staff I met were nice to me**. But I remember one doctor made me sad. She disinfected the chair immediately when I left the clinic. I do get support from my husband and parents. But I can never tell others.” (Woman, with syphilis, 23 years old, middle-school education, local resident, has one baby already, at 23 gestational weeks.)*

The participants also described the use of EMTCT services, and barriers to availing healthcare services.

“*I received my first antenatal care at nine weeks of pregnancy. Although my husband is the sole labor force in the family, I have economic support from my parents. My husband drives me to the doctor every time. **The doctors were so kind and what they said and what they did always warmed me**. I am not sure whether all HIV-infected women have received equal services. Because I met a good doctor here, I appreciate the EMTCT service.” (Woman, with HIV, 29 years sold, middle-school education, first pregnancy, at 24 gestational weeks.)*

“*I knew the result one week after I had HIV screening. My doctor told me that over 95% of babies would not be vertically transmitted if the mother received EMTCT service. **So I sought antiviral therapy immediately at the appointed hospital**. I was always worried that my baby would get infected until he was 18 months old and tested HIV negative. This made me very happy.” (Woman, with HIV positive, 31 years old, middle-school education, already delivered a baby)*

“*I received the first antenatal healthcare service at 12 weeks of gestation. I got most of the health advices from the Internet. I have had one baby already. Money is a barrier for me because I am unemployed. Luckily, **I have rural cooperative medical insurance**. Also, it's only 10 kilometers from my home to the hospital, so **traffic is not a challenge** to me. I would not like to receive ANC at the community hospital, because **I worried about running into my neighbors**.” (Woman, with syphilis, 23 years old, middle-school education, resident, has one baby already, at 23 gestational weeks.)*

## Discussion

In this study, we found that HIV- and syphilis-related stigmatization were common in pregnant women over the past year. Overall, 53.76% of pregnant women experienced HIV-related stigmatization. The proportion was lower, comparative to 79.1% of internalized stigmatization in the United States, 77.8% of overt stigmatization in women from Chile, and 62.2% of anticipatory stigmatization in PLWH receiving ART in the Oromia region, Ethiopia (5, 6, 25); however, our number was found to be greater than the 29.0% of enacted stigmatization in PLWH in South Africa, and 25.2% in depressed pregnant women living with HIV in Kenya, and less than the 10% enacted stigmatization in participants at HIV clinics in Guangxi China (7, 13, 21). Difference in the HIV-related stigma was partially due to variations in social background and culture, participants' characteristics and perspectives toward HIV. Most of the previous studies were conducted in areas of HIV high prevalence or in underdeveloped areas, recorded participant's experiences over the past 12 months, and used expert's scales. Stigmatization is typically regarded as a disorder of individuals who are discreditable (26). Normally, enacted or overt stigmatization appears as discrimination from the living environment or judgment from others; internalized stigmatization reflects the decisions or thoughts of the individuals themselves; and anticipated stigmatization indicates being verbally or physically insulted (6, 7). In the present study, we defined stigmatization as any of the items such as discrimination enacted at home or in the community, workplace, or healthcare institution, without including information on internalized stigmatization or anticipated stigmatization, although, there was evidence of internalized or anticipated stigmatization by qualitative analysis. For example, participants in our study felt ashamed when they discovered about their HIV or syphilis status.

We found that 24.36% of pregnant women with syphilis also experienced stigmatization. During an in-depth interview, one woman with syphilis explained in detail her syphilis-related stigmatization experience. As one of the major sexually transmitted diseases, syphilis has remained a stigmatized disease with a long history (16, 27). A study by Poznan University of Medical Sciences revealed that 75% of women infected with syphilis encountered considerable humiliation, which had a greater proportion than that among women with other STDs; however, the study included only 25 syphilis cases (14). Currently, studies on syphilis-related stigmatization in pregnant women are limited. Syphilis has a much higher endemic rate relative to HIV, therefore it is of critical importance to consider research about syphilis-related stigmatization in pregnant women.

Women with HIV or syphilis exhibited similar socio-demographic characteristics in this study. The majority of women were poorly educated, had higher gravidity and parity, and over half of our study population's partners were also infected. A higher proportion of HIV-infected women came from other provinces and were minorities, similar to previous studies. Some studies have addressed the disparity and required targeting of these vulnerable populations (22, 28). Additionally, sex disparity, incomplete schooling, low income, and depression were frequently reported as driving forces for stigmatization, as these factors might interact and influence each other (7, 9, 20). We have provided integrated HIV and syphilis EMTCT services for pregnant women, and believe that it is of paramount importance to reduce stigmatization toward pregnant women with HIV and syphilis.

Our findings revealed that social support and stigmatization were highly interrelated (6, 18, 19). When stigma was adjusted for maternal characteristics, the risk of poor social support increased by a factor of 2.927. Women with HIV infection faced poor subjective and objective social support compared to those with syphilis, which is likely due to a higher level of stigma attributed to HIV-infected women. HIV-support groups and activities may thus protect women with HIV from encountering stigmatization (7, 22). Additionally, we found that employment may improve social support in accordance with previous studies (29, 30). This information may assist in advocating for, educating, and encouraging women with HIV or syphilis to seek jobs and participate in community activities. In this study, we observed no differences in the support provided by medical staff among women with HIV or syphilis, which may be due to awareness programs in healthcare facilities. Since the initiation of EMTCT, focused communication platforms such as *we chat* groups have been established to advocate health and counseling for pregnant women with HIV or syphilis. EMTCT has also promoted awareness and training against stigmatization for healthcare providers. This helped to reduce stigmatization in healthcare facilities. A study in New Zealand showed that 47% of PLWH experienced HIV-related stigmatization in healthcare settings (31), whereas, 45% of pregnant women with HIV or syphilis received support from medical staff in our study. Additionally, our qualitative research also reflected support from healthcare providers in our study.

To the best of our knowledge, this is the first study initiated to explore comparisons and associations of stigmatization with social support among pregnant women with HIV or syphilis in eastern China. Our study was conducted in a relatively low HIV-prevalence area, which was economically developed, and with a high syphilis prevalence. Support from healthcare providers may have a positive impact on the health of women with HIV or syphilis. There were several limitations in our study. First, three items on stigmatization estimation may limit accurate assessment for subscale stigmatization. Second, some other factors associated with stigmatization were not included, such as health status, floating population, and religious beliefs at the individual or community level (7, 22, 31). Third, factors such as physical functioning, therapy, psychologic distress may account for social support (6, 19, 32), but we were not able to determine a causal link between stigmatization and social support since this was a crosssectional study. Fourth, we defined poor social support as the lowest 25% of total social -support scores with respect to the IQR. This might lead to some measurement bias by leaving out women who lacked social support. Finally, convenience sampling might also have led to a certain selection bias as pregnant women who volunteered for the survey may have been more likely to have received better support or have faced less stigmatization than other women.

## Conclusions

Conclusively, HIV- or syphilis-related stigmatization was significant in pregnant women and it was higher in pregnant women with HIV. Women with HIV experienced poor social support compared to those with syphilis. Stigmatization along with minority ethnicity was negatively associated with social support in women with HIV or syphilis. Employment was associated with improved social support. As support from healthcare providers, with respect to pregnant women with HIV or syphilis did not differ, we posit that support from healthcare providers may positively impact the health of pregnant women with HIV or syphilis. Implementation of strategies to reduce stigmatization and to improve social support should therefore be advocated for women with either HIV or syphilis infection during pregnancy.

## Data Availability Statement

The datasets presented in this article are not readily available because of privacy and ethical concerns. Requests to access the datasets should be directed to XiaZ, zjfb_amy@zju.edu.cn.

## Ethics Statement

The studies involving human participants were reviewed and approved by the Ethics Committee of the Women's Hospital School of Medicine, Zhejiang University. The patients/participants provided their written informed consent to participate in this study.

## Author Contributions

XZ and XinW designed the study. XZ analyzed the data. XZ and XiaW drafted the manuscript. XZ, HW, and XH collected the data. XZ, XiaW, HW, XH, and XinW provided critical comments or revised the manuscript. XinW supervised the study. All authors approved the final version of the article, including the authorship list.

## Funding

This work was supported by Zhejiang Provincial Science and Technology Department [2019C35028]. The study was also funded by UNICEF China, 501 Health, Nutrition and Wash- MCH; increased evidence and policy makers' capacity piloting, elimination of mother to child transmission of HIV, syphilis, and HBV (EMTCT).

## Conflict of Interest

The authors declare that the research was conducted in the absence of any commercial or financial relationships that could be construed as a potential conflict of interest.

## Publisher's Note

All claims expressed in this article are solely those of the authors and do not necessarily represent those of their affiliated organizations, or those of the publisher, the editors and the reviewers. Any product that may be evaluated in this article, or claim that may be made by its manufacturer, is not guaranteed or endorsed by the publisher.

## Abbreviations

MTCT: Mother-to-child transmission
EMTCT: Elimination of mother-to-child transmission
ANC: Antenatal health care.
